# Aspects des traumatismes fermés de l’abdomen opérés à l’Hôpital Général de Référence Nationale de N’Djaména (HGRN), Tchad: à propos de 49 cas

**DOI:** 10.11604/pamj.2017.26.50.8327

**Published:** 2017-01-31

**Authors:** Ouchemi Choua, Kimassoum Rimtebaye, Ngueidjo Yamingue, Kalli Moussa, Mignagnal Kaboro

**Affiliations:** 1 Département de Chirurgie, Faculté des Sciences de la Santé Humaine BP 1117 N’Djaména, Tchad

**Keywords:** Traumatisme abdominal fermé, lésions du grêle, rupture de vessie, accidents de la voie publique, Tchad, Blunt abdominal trauma, lesion of the small intestine, bladder rupture, road traffic accident, Chad

## Abstract

**Introduction:**

Les traumatismes fermés de l’abdomen sont fréquents.

**Méthodes:**

Il s’agissait d’une étude rétrospective sur 49 dossiers de patients opérés pour traumatisme fermé de l’abdomen en cinq ans à l’Hôpital General de Référence Nationale de N’Djaména au Tchad. Les paramètres épidémiologiques, cliniques et thérapeutiques étaient étudiés.

**Résultats:**

C’étaient 42 hommes et 7 femmes d’âge moyen de 21,3 ans. Les étiologies étaient: les accidents de la voie publique dans 61,2% des cas; les écroulements de mur (14,3%); les agressions (8,2%). Les traumatismes fermés de l’abdomen étaient plus fréquents au mois d’Août (14,28%) et Octobre (16,32%). Le délai d’admission à l’hôpital était de 6 à 12h dans 43% des cas. Le moyen d’évacuation des blessés était une voiture privée dans 85,7% des cas. Cliniquement, l’état hémodynamique était souvent stable (55,1%). L’imagerie médicale était dominée par la radiographie directe de l’abdomen (57,1%). Les lésions les plus observées ont été celles du grêle seul (16,32%) ou associées à celle de la vessie (8,16%), et de la rate (2,04%). La laparotomie était négative dans 6,12% des cas. La morbidité (12,2%) était dominée par les abcès de paroi. Le taux de décès était de 6,1%.

**Conclusion:**

Les accidents de la voie publique sont la première cause de traumatismes fermés de l’abdomen. Le délai diagnostic et thérapeutique est important. Des mesures de sécurité routière devraient prévenir les accidents.

## Abstract (en)

**Introduction:**

Blunt abdominal traumas are common.

**Methods:**

We retrospectively reviewed the medical records of 49 patients with blunt abdominal trauma who underwent surgery at the General Hospital of National Reference of N’Djamena, Chad over a period of 5 years. Epidemiological, clinical and therapeutic parameters of patients were studied.

**Results:**

The study included 42 men and 7 women, mean age 21.3 years. The causes of blunt abdominal traumas were: road traffic accidents in 61.2% of cases; wall collapses (14.3%); assaults (8.2%). Blunt abdominal traumas were more frequent in August (14.28%) and October (16.32%). The waiting time for admission in hospital was 6-12h in 43% of cases. At discharge, wounded patients used private car in 85.7% of cases. Clinically, patients were often hemodynamically stable (55.1%). Medical imaging was dominated by direct radiography of the abdomen (57.1%). The most observed lesions were those located only in the small intestine (16.32%) or related to that of the bladder (8.16%) and spleen (2.04%). Laparotomy was negative in 6.12% of cases. Morbidity (12.2%) was dominated by abdominal wall abscess. Mortality rate was 6.1%.

**Conclusion:**

Road traffic accidents are the leading cause of blunt abdominal traumas. It is important to minimize delays in diagnosis, and treatment. Road safety measures should be implemented to prevent accidents.

## Introduction

Les traumatismes abdominaux fermés encore appelés contusions abdominales sont des lésions produites au niveau de l’abdomen, de son contenu ou de ses parois, par un traumatisme ayant respecté la continuité pariétale. Ils peuvent survenir de façon isolée (accident de sport, agression), ou plus fréquemment, dans le cadre d’un polytraumatisme (accident de la voie publique) [1]. Ils constituent 70 à 86% des traumatismes abdominaux. Leur taux de mortalité est de l’ordre de 20%, lié à la gravité des lésions uniques d’une part, et d’autres parts, à un contexte de polytraumatisme [1, 2]. Des lésions d’organes creux par choc direct et hyperpression ont été observées chez les traumatisés ayant porté la ceinture de sécurité. Cependant, le port de ceinture de sécurité a permis de réduire de 40 à 50% le taux de mortalité depuis 2005 en France [3]. Le but de ce travail était d’étudier les aspects épidémiologiques, cliniques et thérapeutiques des traumatismes fermés de l’abdomen à l’Hôpital Général de la Référence Nationale de N’Djamena.

## Méthodes

Il s’agissait d’une étude rétrospective allant du 1^er^ Janvier 2009 au 31 Décembre 2013 menée dans le Service de Chirurgie Générale de l’Hôpital Général de Référence Nationale de N’Djamena (HGRN). Les données étaient recueillies à partir du registre du bloc opératoire et des dossiers cliniques des patients opérés pour traumatisme fermé de l’abdomen. Les variables étudiées étaient: l’âge, le sexe, les causes, le mois, le moyen d’évacuation vers l’établissement sanitaire, le délai d’admission, la durée d’hospitalisation, les moyens de diagnostic, les organes lésés et les lésions associées, les complications et le devenir des patients. La saisie et l’analyse des données ont été effectuées sur le logiciel Word, Excel 2007 et Statistical Package and Social Science Windows 16.0. L’analyse statistique a consisté au calcul des différentes fréquences des variables étudiées. Certaines ont été comparées en utilisant le test Khi^2^ et P<0,05.

## Résultats

Du 1^er^ Janvier 2009 au 31 Décembre 2013 (60 mois), 49 dossiers de contusions abdominales opérées étaient recensés et retenus. Les contusions abdominales représentaient 5,18% des 946 urgences abdominales et 2,13% des 10437 interventions chirurgicales. Il s’agissait de 42 hommes (85,7%) et 7 femmes (14,3%). Le sex ratio était de 6/1. La tranche d’âge la plus touchée était celle de 20 à 29 ans (34,7%). L’âge moyen était de 21,3 ans avec des extrêmes de 4 ans et 35ans. Nous avons constaté que les contusions abdominales étaient plus fréquentes au mois d’Octobre (16,32%) et au mois d’Août (14,28%). Les causes de contusions abdominales opérées sont rapportées dans le Tableau 1. Au cours des accidents de la voie publique, une moto était impliquée dans 61% des cas. Les passagers étaient concernés dans 25 cas sur le total des 49 traumatismes fermés de l’abdomen (51%), tandis que les piétons étaient concernés dans 4 cas (8,2%). Le mécanisme lésionnel était une projection contre la chaussée ou contre un des engins impliqués dans l’accident au cours des accidents de la voie publique. Il impliquait un choc direct dans les écroulements de murs, et dans les coups et blessures volontaires. Le transport des patients à l’hôpital était effectué par une voiture de particulier ou un taxi dans 42 cas (87,5%); un véhicule de police dans 5 cas, une ambulance non médicalisée chez deux patients (4,1%). Tous les patients (100%) avaient présenté une douleur, trois d’entre eux avaient une fièvre à plus de 38,5° tandis que 22 patients (18,4%) avaient des vomissements. L’examen physique retrouvait un tableau de péritonite caractérisé par une défense abdominale dans 36 cas (74,1%). L’état hémodynamique des patients était stable dans 27 cas (55,1%) et instable dans 22 cas (44,9%). Le taux d’hémoglobine était inférieur à 9gr dans 75,5% des cas et chez 24 patients (49%), cela était lié à un hémopéritoine.

**Tableau 1:** Causes de traumatismes fermés de l’abdomen

cause	Effectifs (N)	Fréquence (%)
Accident de la voie publique	30	61,2%
Ecroulement de mur	8	16,32%
Chute d’une hauteur	8	6,32%
Coups et blessures volontaires	4	8,16%
Coups et blessures volontaires	4	8,16%
**Total**	49	100%

Des examens d’imagerie médicale étaient réalisés chez 29 patients (57,15%). Il s’agissait d’une radiographie chez 20 patients (40,85%), d’une échographie chez 7 patients, et d’une échographie associée à une radiographie dans 2 cas. Les radiographies étaient toutes réalisées quand il y’avait une suspicion de fractures. L’échographie avait retrouvé dans tous les cas un épanchement intrabdominale qui s’associait dans 2 cas à une lésion de la rate. Délais dans la prise en charge: 39 patients (79,6%) étaient reçus dans un intervalle de moins de 6 heures après le traumatisme. Cependant 21 patients au total avaient fait au moins 2 fois le parcours de leur domicile à une consultation médicale dans les 24 heures après l’accident. Le premier accès à l’hôpital ne retrouvait pas de signes de gravité. Dans 1 cas, le patient avait initialement refusé l’intervention chirurgicale proposée. Il fut opéré après 34 heures. Le retard moyen global était de 11 heures. Tous les blessés avaient bénéficié d’une laparotomie médiane sus et sous ombilicale. Le Tableau 2 reporte les lésions intra abdominales. Les actes chirurgicaux réalisés étaient faits de: 20 sutures du grêle; trois résections-anastomoses termino-terminale du grêle, dont une était associée à une résection de la queue du pancréas. Par ailleurs nous avions réalisé 9 splénectomies et une suture de la rate. Une néphrectomie totale droite était pratiquée à cause d’un traumatisme stade IV dans un contexte de poly traumatisme, avec notamment une fracture du bassin. Une invagination intestinale était réduite. Dans 17 cas les lésions des organes intra abdominaux avaient provoqué un hémopéritoine d’environ 800 cc de sang en moyenne. Dans trois cas (6,12%), la laparotomie ne retrouvait pas de lésions. Les associations lésionnelles extra abdominales sont regroupées dans le Tableau 3.

**Tableau 2:** Répartition des lésions intra abdominales+

Lésions d’organe	Effectif (N)	Fréquence (%)
Grêle	23	50 %
Rate	10	21,8 %
Lésions vasculaires	6	13 %
Vessie	3	6,5 %
Foie	2	4,3 %
Rein	1	2,2%
Pancréas	1	2,2%
Total	46	100%

+ Les diverses lésions pouvaient être associées entre elles

**Tableau 3:** Lésions extra abdominales associées aux traumatismes fermés de l’abdomen

Siège des lésions	Effectif (N)	Fréquence (%)
Fracture du bassin	2	28,6 %
Fracture du col du fémur	2	28,6 %
Fracture de cote	1	4,28 %
Fracture vertébrale stable	1	14,28 %
Contusion pulmonaire	1	14,28 %
Total	7	100%

La durée moyenne d’hospitalisation était de 9,2 jours. Une suppuration pariétale était retrouvée chez 6 patients, soit 12,24%, tandis que des complications médicales en l’occurrence un épanchement pleural gauche et une pneumonie basale droite avaient émaillé le décours postopératoire de 2 patients (4,1%). Trois patients étaient décédés. Il s’agissait d’un patient admis pour hémopéritoine suite à une contusion hépatique; d’un cas de traumatisme grave du rein droit ayant comporté une néphrectomie et d’un patient opéré pour perforation post traumatique du grêle. Tous les décès étaient intervenus entre J1 et J2 postopératoires. Il s’agissait toujours de traumatismes fermés de l’abdomen des suites d’un accident de la voie publique par un mécanisme de projection ou de collision. Il n’y avait pas de corrélation significative entre type d’accident et décès (Khi^2^ =2,024; P=0,996).

## Discussion

Les traumatismes fermés de l’abdomen concernent le jeune adulte de sexe masculin. La moyenne d’âge de notre série est de 21,3 ans, avec un sex ratio de 6 hommes pour une femme. D’autres séries trouvent une moyenne d’âge de 19 à 31,8 ans et un sex ratio similaire au notre [1, 4, 5]. Dans notre contexte les hommes jeunes sont les principaux utilisateurs de motocyclettes, engins les plus impliqués dans les accidents de la voie publique [6]. La prédominance des accidents de la voie publique est une constante dans la sous-région: au Nigeria, en RDC, et au Cameroun [7–9]. Les causes sont multifactorielles: augmentation exponentielle du nombre de voitures et de motos dans les pays émergents, inobservance des normes élémentaire de sécurité routière. Par ailleurs l’état des routes, leur illumination nocturne, et la promiscuité entre divers types d’engins motorisés, ou d’animaux de somme et de piétons, augmentent l’exposition aux accidents [10]. Les mois les plus pluvieux de l’année sont aussi les plus grands pourvoyeurs de contusions abdominales. En effet, aux victimes des accidents de la voie publique, viennent s’associer les traumatisés par écroulement de murs des maisons. Les habitations de certains quartiers périphériques de N’Djaména sont construites en briques de banco, qui s’effritent suite aux infiltrations d’eau dans les zones du lit du fleuve Chari. Il se pose donc un problème d’aménagement des structures de drainage des eaux de pluies, dans les faubourgs de la capitale.

Dans 85% des cas de notre série le moyen d’évacuation des blessés à l’hôpital est un véhicule privé (taxi, voiture de particulier). Une ambulance, par ailleurs non médicalisée transporte seulement 3% des patients. Nous pouvons supposer que seuls les malades les plus stables arrivent jusqu’au service des urgences. A Madagascar, Raheri et al [11] constatent en effet que l’autopsie des blessés à traumatisme fermé de l’abdomen révélait un hémopéritoine moyen de 2,5 l, des ruptures hépatiques ou spléniques, et des plaies vasculaires rétropéritonéales. La majorité des patients de notre série (76,9%) arrive à la consultation médicale dans un intervalle de 0 à 6 heures. Le délai moyen est cependant élevé dans notre série (11heures) à cause du diagnostic tardif, et une irritation péritonéale franche est retrouvée dans 74,1% des cas. C’est le cas aussi dans d’autres séries africaines [11–14]. Les raisons sont multiples: sous estimation des symptômes au premier passage à l’hôpital, absence de moyens de transport, recours à la médecine traditionnelle. Un état de choc hypovolémique est décrit dans 44,9% de nos cas, et 45,4% dans la série de Méhinto et al [15]. Ces patients manquent de toute prise en charge pré hospitalière.

La perforation de l’intestin grêle est la lésion la plus fréquente de notre série, suivie par larupture de la rate. Cela est à mettre en relation avec le type d’accident, impliquant très souvent des motocyclettes, dont le guidon peut contusionner directement l’abdomen. Le mécanisme de lésion est constitué par l’écrasement, l’éclatement des organes, ou par l’effet de la décélération. Ces différents mécanismes peuvent agir seuls ou de manière diversement associés [9]. Les perforations du grêle sont décrites dans 3% à 5% des traumatismes fermés de l’abdomen [16]. Les lésions isolées du grêle sont rares; environ 0,3% selon Watts et al [17] qui ont étudié plus de 275000 lésions des viscères creux dans les traumatismes fermés de l’abdomen. Il s’agit en général de lésions typiques des séries pédiatriques ou de jeunes adultes. Le mécanisme incriminé est la chute d’une hauteur, ou le traumatisme de guidon de motocyclette [18–21]. Chez l’adulte ce type de traumatisme peut être provoqué par la ceinture de sécurité [18, 22].

Nous ne le constatons pas dans notre série où les passagers d’autos n’ont pas reporté de blessures intestinales. La rate est l’organe plein le plus touché; nous procédons à 8 splénectomies totales et une suture capsulaire de la rate. Dans tous ces cas, le choc hypovolémique et la réaction pariétale abdominale nous obligent à intervenir rapidement, d’autant plus que les examens radiologiques d’orientation sont difficilement praticables en urgence (échographie, scanner). En littérature 60 à 80% des adultes présentant un traumatisme de rate ne sont pas splénectomisés [1, 9, 23, 24]. Le meilleur taux de sauvetage splénique est obtenu par le traitement non opératoire. Il doit se faire en milieu de réanimation chirurgicale. Les conditions requises sont une stabilité hémodynamique, une absence de suspicion de perforation d’organe creux, et la disponibilité des examens morphologiques [24, 25]. Il faut cependant souligner que même dans les pays à ressources limités comme au Maroc, le traitement conservatoire peut être efficacement réalisé. En effet, au prix d’un examen clinique bi-quotidien, d’un hémogramme quotidien, d’une échographie abdominale répétée à 48 heures et d’un scanner à la demande, le taux de sauvetage de la rate est de 80% [23, 24]. Ce taux est proche de celui des grands Trauma Centers aux Etats Unis ou en France où sont pratiqués des scanners systématiques et des embolisations spléniques précoces [23, 25–27]. Nous devrions donc insister même dans notre contexte sur la pratique d’un examen clinique répété, sur l’importance de l’échographie et la connaissance des facteurs pronostics dans le traitement non opératoire des traumatismes de la rate. Dans nos conditions actuelles d’exercice, beaucoup d’examens complémentaires sont difficilement réalisables par manque de plateau technique et humain.

Parmi les lésions associées à celles de l’intestin grêle et de la rate, la plus fréquente est la rupture intra-péritonéale de la vessie. Quand elle est isolée, cette lésion est de diagnostique difficile parce que rare. Selon Wirth et al [28] seulement 17% des ruptures post-traumatiques de la vessie sont isolées comme dans deux des cas de notre série. Souvent, la rupture intrapéritonéale de la vessie qui constitue 25% à 43% des ruptures post-traumatiques de cet organe est associée à d’autres lésions, surtout des fractures de la ceinture pelvienne [29]. Notre traitement est une suture de la brèche vésicale au Vicryl résorbable 2.0, en double plan, en points séparés. Chez les patients hémodynamiquement stables la réparation peut se faire également en laparoscopie [29]. Le taux de laparotomies blanches de notre série est de 6,12%; il varie en littérature de 5% à 16,6% [11, 13]. La durée d’hospitalisation est longue, en moyenne 9,2 jours, en rapport avec les complications pariétales par inoculation de liquide péritonéal. Le taux de décès est de 6,12%, comparable à celui de travaux de pays en développement, et de certaines séries occidentales [12–15, 18, 20–21]. Le retard dans la prise en charge et la gravité des lésions semble corrélé à la mortalité. En effet les trois cas de décès de notre série concernent une péritonite par perforation du grêle opérée après 24 heures, un polytraumatisé avec une désinsertion de l’hile rénale gauche, et une contusion hépatique grave. La compromission de ces malades graves a lieu dans la phase pré hospitalière ou en intraopératoire par la survenue d’un cercle vicieux associant acidose métabolique, hypothermie et coagulopathie.

## Conclusion

Les traumatismes fermés de l’abdomen ont une fréquence élevée dans la population jeune et de sexe masculin, souvent victime d’accident de la voie publique. Le long délai d’admission et la difficulté d’accessibilité à l’imagerie dans notre service des urgences justifient la pratique courante de laparotomie à visée diagnostique et thérapeutique.

### Etat des connaissances actuelles sur le sujet

- Les contusions de l’abdomen concernent surtout le jeune adulte de sexe masculin;
- Les accidents de la voie publique sont la circonstance de survenue plus fréquente.

### Contribution de notre étude à la connaissance

- Le mécanisme de traumatisme abdominal par guidon de motocyclette est le plus fréquent;
- Les perforations de l’intestin grêle sont les plus fréquentes. Le diagnostic est souvent tardif et conditionné par une péritonite.
